# Does Depth of Breathing Matter in Gastroesophageal Reflux? Esophagogastric Junction Barrier Function Should Also Be Considered

**DOI:** 10.1111/den.70038

**Published:** 2025-09-17

**Authors:** Shiko Kuribayashi, Toshio Uraoka

**Affiliations:** ^1^ Department of Gastroenterology and Hepatology Gunma University Graduate School of Medicine Maebashi Japan

**Keywords:** depth of breathing, endoscopic pressure study integrated system, esophagogastric junction barrier function, gastroesophageal reflux, transdiaphragmatic pressure gradient

## 1 Editorial

Gastroesophageal reflux disease (GERD) is defined as the presence of symptoms or complications related to the reflux of gastric contents into the esophagus. The diagnosis of GERD is primarily made by evaluating the symptoms in clinical practice. When the diagnosis of GERD is inconclusive, esophageal physiological evaluation can show evidence of the presence of GERD. The Lyon consensus provides conclusive criteria for and against the diagnosis of GERD, and the updated Lyon consensus shows that hiatal hernia evaluated by esophagogastroduodenoscopy (EGD) is considered supportive evidence for GERD [1].

The esophagogastric junction (EGJ) consists of the lower esophageal sphincter and the crural diaphragm, which prevent gastroesophageal reflux (GER). This antireflux barrier function of the EGJ has been assessed on EGD. Hill et al. showed that the appearance of a gastroesophageal flap valve (GEFV) was a better predictor of the presence or absence of GER than EGJ pressure [2]. Distending the stomach by air insufflation on EGD could simulate during the postprandial state in which GER is most likely to occur. Therefore, it is reasonable to evaluate the antireflux barrier function of the EGJ during gastric distention on EGD. Although a grading system for GEFV has been proposed, this system is a qualitative evaluation [2]. Thus, a quantitative evaluation of the antireflux barrier function of the EGJ on EGD is warranted.

An endoscopic pressure study integrated system (EPSIS) was developed. EPSIS can evaluate gastroesophageal barrier function quantitatively by measuring intragastric pressure and endoscopically observing the morphology of the EGJ simultaneously [3]. An increase in intragastric pressure by carbon dioxide (CO_2_) insufflation into the stomach is observed when the EGJ antireflux barrier function is preserved (uphill pattern). The intragastric pressure waveform does not show the uphill pattern because CO_2_ evacuates from the stomach continuously when the EGJ barrier function is weakened (flat pattern). The flat pattern and low maximum intragastric pressure during CO_2_ insufflation are associated with GERD. In addition, the pressure difference between the basal intragastric pressure and the maximum intragastric pressure during CO_2_ insufflation and the pressure gradient of the waveform are also related to the acid exposure time (AET) [4]. Recently, it has been reported that EPSIS can assess the dynamics of antireflux barrier function as well as quantitative evaluation [5].

When intragastric pressure is measured during EPSIS, fluctuations in intragastric pressure are observed. Abiko et al. focused on the relationship between the magnitudes of fluctuations in the intragastric pressure during EPSIS and AET measured by esophageal impedance‐pH monitoring and found that the magnitude of pressure fluctuation of the gastric pressure during EPSIS may be related to AET in patients with GERD [6]. Fluctuations of intragastric pressure could be caused by respiratory cycles, vascular pulsations, artifacts, and so on. Based on the cycles of fluctuations evaluated in the study, they must be produced by respiratory cycles. Intragastric pressure increases during inspiration, while it returns to the baseline pressure at the end of expiration, which produces fluctuations in intragastric pressure. The magnitude of wave‐high fluctuations of intragastric pressure may be mainly affected by the depth of breathing and other factors, such as gastric accommodation.

Importantly, esophageal pressure decreases during inspiration, which reflects intrathoracic pressure against the pressure increase of the intragastric pressure. The transdiaphragmatic pressure gradient (TPG) between decreased esophageal pressure and increased intragastric pressure during inspiration is considered to be a cause of gastroesophageal reflux (GER). Since the magnitude of height fluctuation in waveforms during EPSIS reflects the depth of breathing, the results of Abiko, et al.'s study could be interpreted as the magnitude of TPG associated with AET.

TPG has been highlighted as an important factor in the pathogenesis of GERD, especially in sleep studies. When an obstructive sleep apnea (OSA) event occurs, the magnitude of negative pressure in the esophagus and positive pressure in the stomach gradually increases, which is caused by increases in respiratory efforts during the OSA event. Since the prevalence of GERD in patients with OSA is high, it has been thought that this increase in the magnitude of TPG induces a GER event. In addition, continuous positive airway pressure reduced GER events in patients with OSA [7], which supported the idea that increased TPG could induce GER. However, the barrier function of the EGJ is not considered in this theory. We simultaneously performed high‐resolution esophageal manometry, esophageal impedance‐pH monitoring, and polysomnogram during the nighttime in patients with OSA and found that the EGJ acts to prevent GER events against increased TPG [8]. In addition, none of the OSA events had evidence for GER on either impedance or pH recordings. Therefore, the increased magnitude of TPG by augmented respiratory efforts is unlikely to induce GER when the EGJ antireflux barrier function is preserved.

In our study, patients with large hiatal hernia (in which the barrier function of the EGJ was impaired) were not enrolled [8]. The crural diaphragm is responsible for preventing GER during abdominal strain, cough, and increased respiratory effort [9]. Therefore, it is unknown whether augmented TPG could induce GER in patients with large hiatal hernia. Since EGJ barrier function and the magnitude of wave fluctuation in waveforms (reflecting the depth of breathing in EPSIS) can be evaluated with EPSIS, it may be possible to clarify whether TPG is related to GERD, especially in patients with impaired EGJ barrier function. Moreover, restoration of EGJ barrier function by endoscopic antireflux treatment should be effective in these patients. Moreover, evaluation of EGJ barrier function and the magnitude of wave height fluctuation in waveforms should be useful when selecting patients suitable for endoscopic antireflux treatment.

Esophageal manometry and reflux monitoring, which are invasive and less tolerated, must be performed simultaneously during postprandial and sleep states to determine whether TPG could induce GER in impaired EGJ function patients in the recumbent position. To assess EGJ barrier function and depth of breathing simultaneously, it is easier and less invasive to evaluate both EGJ barrier function and the magnitude of wave fluctuation in waveforms in EPSIS.

Obesity, OSA, respiratory diseases (such as COPD), and cardiovascular and neuromuscular diseases are known risk factors that affect the magnitude of TPG in patients. However, the presence of these diseases was not assessed in the study, although the body mass index was evaluated. Moreover, the presence of abdominal breathing could also be a potential factor. The depth of sedation and the presence of airway obstruction by endoscopy could affect the magnitude of the TPG. However, the depth of sedation and the presence of airway obstruction during EPSIS were not assessed. These factors should be considered to standardize this evaluation method for the magnitude of fluctuation in intragastric pressure.

When gas in the stomach is ventilated (in a gastric belch), the upper esophageal sphincter (UES) must be relaxed. Although the UES barrier function is important in patients with the flat pattern in EPSIS, the UES function could not be assessed in EPSIS. Physicians should assess esophageal barrier function in GERD using EPSIS.

Several factors are involved in the pathophysiology of GERD. Although the barrier function of the EGJ is important for the occurrence of GER, transient lower esophageal sphincter relaxation (TLESR) is the main mechanism of GER [10]. Moreover, low LES pressure, swallow‐associated LES relaxation, and strain during periods of low LES pressure are known as other mechanisms of GER. The number of TLESR with acid reflux, esophageal clearance, ability of saliva secretion, acid pocket, and gastric atrophy could affect the value of AET. In addition, esophageal sensation is also an important factor in the pathophysiology of GERD. These factors should be evaluated to manage GERD.

In summary, the magnitude of wave height fluctuation in waveforms during EPSIS may be useful for predicting prolonged AET, especially in patients with impaired EGJ barrier function. Evaluating both the EGJ barrier function and the magnitude of wave height fluctuation in waveforms is important when EPSIS is performed.

## Author Contributions

S.K. was responsible for drafting the manuscript. T.U. was responsible for supervising the manuscript preparation.

## Conflicts of Interest

The authors declare no conflicts of interest.

## Linked Articles

S. Abiko, Y. Shimamura, H. Inoue, et al. Wave Height Fluctuations in the Waveforms of an Endoscopic Pressure Study Integrated System Have the Potential to Predict Acid Reflux in Gastroesophageal Reflux Disease (With Video). https://doi.org/10.1111/den.15049
